# We Don't Talk Enough About Overuse Injuries in Clinicians Using POCUS

**DOI:** 10.24908/pocus.v8i1.16075

**Published:** 2023-04-26

**Authors:** Traci Fox, Kaylah Maloney, Arthur Au, Resa E Lewiss

**Affiliations:** 1 Department of Medical Imaging & Radiation Sciences, Jefferson College of Health Professions Philadelphia , PA; 2 Robert Wood Johnson University Hospital/Rutgers Health New Brunswick, NJ; 3 Thomas Jefferson University Hospital Philadelphia, PA

**Keywords:** POCUS, ergonomics, RSI, injury, WRMSD Letter

## Letter

The hospital is full of clinicians working with poor ergonomic form. In the emergency department, for example, this includes and is not limited to clinicians bending over to place IVs and intubating patients. When it comes to ultrasound, some clinicians might think, “I’m only scanning a few patients a day,” and the examinations are typically quick and focused. Enter the term “repetitive” stress injury” (RSI). To date, the literature pertaining to RSI and point of care ultrasound (POCUS) clinicians is very limited. We think it’s time for this to change.

Work Related Musculoskeletal Disorders (WRMSDs) are insidious, and the symptoms typically occur over time. Whether called WRMSDs, RSI, repetitive motion injuries, overuse syndrome, or cumulative trauma, it's all mostly preventable. Since the literature for POCUS and RSI is limited, we focus on the evidence for physicians who perform ultrasound, also known as sonologists.

The statistics are grim. People performing ultrasound 40 hours a week will have pain within five years. In research specific to sonographers (ultrasound technologists), it is reported that up to 90% of sonographers in practice are scanning in pain. Unsurprisingly, approximately 20% of sonographers will leave the profession, due to musculoskeletal issues 1, 2. This attrition comes at a cost of over $150,000 per sonographer lost to injury. 3, 4

Three common causes of RSI are poor posture, poorly positioning the patient and the equipment, and a poor hand grip. A significant amount of RSI occurs in the trapezius muscle region from non-ergonomic positioning. Relatedly, the most common anatomic areas for RSI are the shoulders, followed closely by the neck 1. Routine scanning requires sonologists to spend a lot of time in a stretching or reaching position. The sonologists may also have injury to their wrists and hands, including carpal tunnel injury, from repeatedly holding the transducer incorrectly. Although ultrasound transducers have been designed to be more ergonomic, the sonologists must avoid unnatural hand positions and achieve a proper grip. This takes instruction and practice. New learners of ultrasound often do not change their grip as the transducer moves around, and tend to hold the transducer tightly. With too firm a grip, the sonologist loses the fine motor skills needed to perform the ultrasound. Muscles then fatigue faster. Finally, injury to the lower back is also common due poor posture and to not adjusting the height of the bed. 

On a positive note, there are preventive measures, that can be done, to reduce the RSI risk. Sonologists can greatly reduce risk by monitoring body and equipment position and doing shoulder and wrist stretches during the shift. When practiced consistently, every case and over time, permanent injury can be mitigated 1, 2.

The National Institute for Occupational Safety and Health (NIOSH) and other organizations have an abundance of recommendations to improve scanning ergonomics 5. Two resources with educational videos include Healthy Sonographer (https://healthysonographer.com/), a website by Canon (Canon Medical Systems, USA) with explanations and videos of improving ergonomics in ultrasound 6, and The Society of Diagnostic Medical Sonography (SDMS.org; Plano, TX) 2. 

Unfortunately, the reality is that sonologists use awkward arm positions to obtain some views, and a lack of ergonomic equipment often results in overreaching while twisting the back. This leads to lower back and neck pain. Proper ergonomics and preventative stretches take time - something that is scarce in all of medicine. There are adjustments and adaptations that can be done to improve ergonomics and prevent RSI. Every ultrasound examination should begin with the following introspection: Is the patient close enough to me? Am I going to need to reach/extend? Did I adjust the chair or the bed height? 

**Figure 1:**
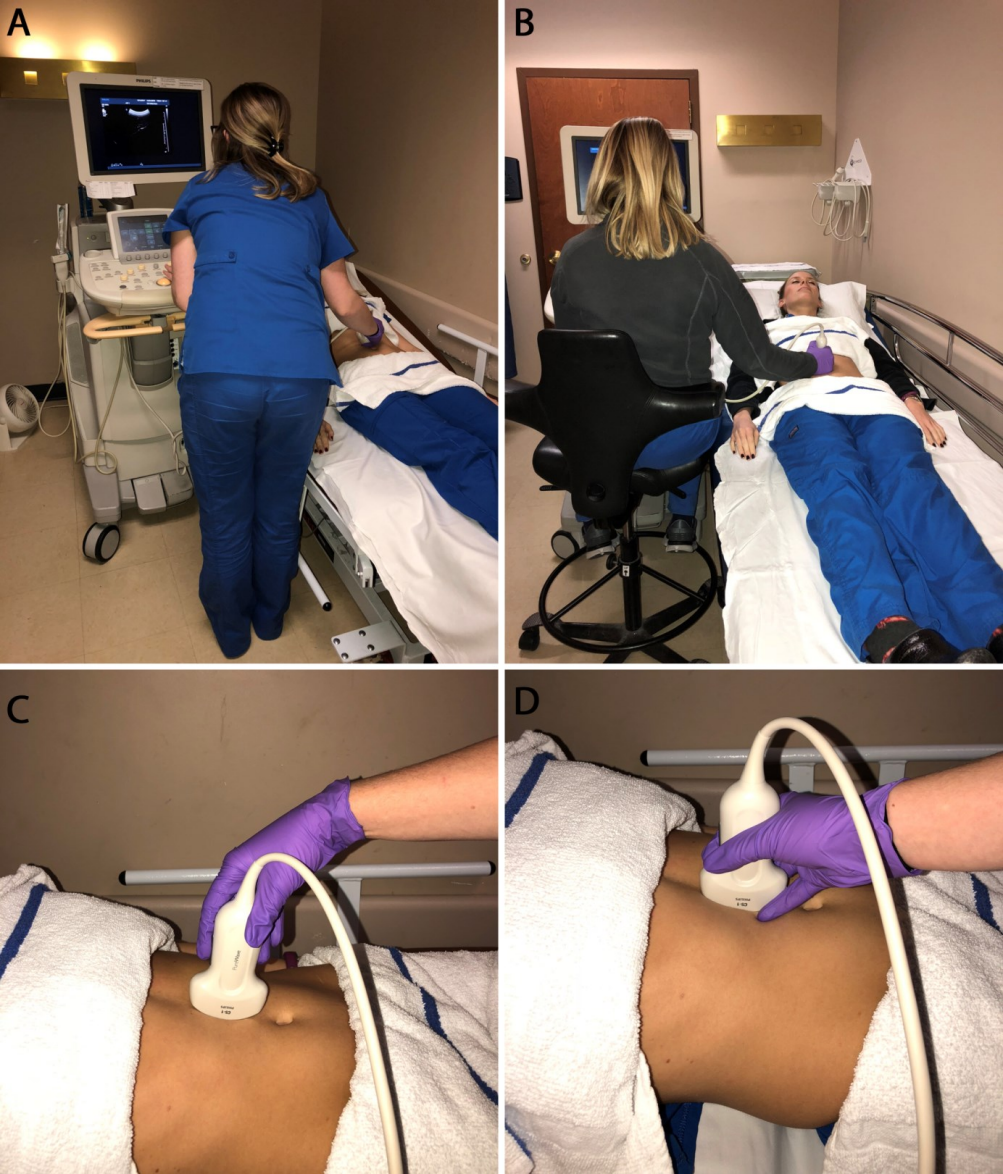
A) Incorrect positioning. The sonologist is leaning forward. The stretcher is too low. The stretcher should be raised or the sonologist should sit. B) Correct positioning. The sonologist is seated at a height that allows for upright back and arm with minimal abduction. Her eyes are level with the monitor. C) Incorrect transducer grip. The transducer is held too high and with a flexed wrist. This prevents transducer control and places the sonologist at risk for carpal tunnel disorders. D) Correct transducer grip. The transducer is held low with fingers touching the patient (anchored). The wrist is straight and not awkwardly flexed or extended. (Images with permission by Davies Publishing)

Here we list a few preventive measures:

- Learn to be ambidextrous and intentionally avoid reaching and twisting.
- Use a chair. This may help if the table or stretcher cannot change height (Figure 1).
- Use a bedside step stool to avoid having to lift your arm too high.
- Use a foam sponge that can be used to rest the arm on for support.
- Prevent overreaching and over abducting: keep the arm stretch within 30 centimeters of the body and the angle of the arm within 30 degrees.
- Create a “magic triangle”, which means that the patient and control panel should be within arm’s reach, with minimized reaching and little or no body lean.
- Hold the transducer firmly enough that pressure can be applied to the patient when needed.
- Avoid awkward rotation of the hand when holding the transducer and keep a firm (but not too firm) grip.
- Hold the transducer low - having at least one finger anchored on the patient is crucial for tactile feedback to determine if the transducer is sliding off the area of interest and how much pressure is being applied (Figure 1).
• Use a cable brace to support the transducer cable and ensure it is not getting in the way and relieve the weight of the cord 5.

One of the limitations of ergonomics is that manufacturers tout the ergonomic features of their equipment; however, those very same features are sometimes only found on the highest priced full-size ultrasound machines. Ergonomic stretchers and/or chairs are expensive. The cost could arguably be offset by showing administrators the cost of improving ergonomics compared to the financial loss from losing clinicians. While it is prudent that providers are consciously working to improve their daily ergonomics, it is equally important for their institution to minimize ergonomic stressors. Practicing proper ergonomics must be performed with every patient, every day, or providers will get hurt. By the time that person has symptoms, the damage is already done.

The time is now for physician practitioners of ultrasound to improve ergonomics and reduce absence due to injury from WRMSDs or RSIs 7. We know that education and re-education is important 3. In fact, we suggest that RSI should be an annual and mandatory compliance training for every POCUS program.

## Disclosures

KM received honoraria for ACEP ultrasound workshop lectures. REM serves on the Medical Advisory Board for EchoNous Inc. 
